# Supplementary material for: Hysterectomy for Benign Indications and Risk of Cataract Formation in South Korean Women
Source: Medicina (Kaunas). 2023 Sep 8;59(9):1627. doi: 10.3390/medicina59091627 (PMC10538100; doi:10.3390/medicina59091627)
Supplement: Supplementary file 1 [file medicina-59-01627-s001.zip › Supplementary_Table S1.pdf]

Supplementary Table S1. Case/person-years for cataracts in participants with or without hysterectomy (National Health Insurance Database, 2007–2020)

	Non-Hysterectomy	Hysterectomy	Hysterectomy + adnexal surgery
Total	1,787/663,863 (269)	1,235/523,966 (236)	413/150,612 (274)
Age at inclusion (years)			
40~44	243/252,239 (96)	175/166,750 (105)	36/38,788 (93)
45~49	505/227,081 (222)	478/227,159 (210)	136/58,174 (234)
50~54	717/151,008 (475)	442/112,155 (394)	164/42,351 (387)
50~55	322/33,534 (960)	140/17,902 (782)	77/11,299 (682)
SES			
Mid to high SES	1,710/645,210 (265)	1,195/509,228 (235)	397/146,638 (271)
Low SES	77/18,652 (413)	40/14,738 (271)	16/3,974 (403)
Region			
Urban area	1,105/378,433 (292)	794/310,224 (256)	284/96,993 (293)
Rural area	682/285,430 (239)	441/213,742 (206)	129/53,619 (241)
CCI			
0	1,113/492,242 (226)	809/387,409 (209)	250/108,950 (229)
1	361/99,976 (361)	252/78,130 (323)	92/23,798 (387)
≥2	313/71,644 (437)	174/58,427 (298)	71/17,863 (397)
Parity in cohort			
0	1,777/655,517 (271)	1,230/516,946 (238)	412/149,087 (276)
1	8/6,035 (133)	3/4,940 (61)	/1,119 (0)
≥2	2/2,311 (87)	2/2,080 (96)	1/406 (246)
Adnexal surgery before inclusion			
Absent	1,776/654,917 (271)	1,227/518,800 (237)	409/147,361 (278)
Present	11/8,945 (123)	8/5,166 (155)	4/3,251 (123)
Hypertension before inclusion			
Absent	1,292/557,367 (232)	946/437,784 (216)	270/120,586 (224)
Present	495/106,496 (465)	289/86,182 (335)	143/30,025 (476)
DM before inclusion			
Absent	1,442/607,675 (237)	1,005/478,121 (210)	317/135,209 (234)
Present	345/56,188 (614)	230/45,845 (502)	96/15,403 (623)
Dyslipidemia before inclusion			
Absent	1,210/531,806 (228)	887/418,870 (212)	249/115,901 (215)
Present	577/132,056 (437)	348/105,096 (331)	164/34,711 (472)
Menopause before inclusion			
Absent	1,385/590,053 (235)	1,001/470,903 (213)	317/130,000 (244)
Present	402/73,810 (545)	234/53,036 (441)	96/20,611 (466)
MHT before inclusion			
Absent	1,729/654,675 (264)	1,194/515,108 (232)	402/147,283 (273)
Present	58/9,188 (631)	41/8,858 (463)	11/3,329 (330)
MHT after inclusion			
Absent	1,647/614,637 (268)	1,014/446,645 (227)	326/117,650 (277)
Present	140/49,226 (284)	221/77,321 (286)	87/32,962 (264)

CCI, Charlson comorbidity index; DM, diabetes mellitus; MHT, menopausal hormone therapy; SES, socioeconomic status. Data are expressed as the case/person-years (case/100,000 person-years).
