# Supplementary figures and images for: Hysterectomy for Benign Indications and Risk of Cataract Formation in South Korean Women
Source: Medicina (Kaunas). 2023 Sep 8;59(9):1627. doi: 10.3390/medicina59091627 (PMC10538100; doi:10.3390/medicina59091627)

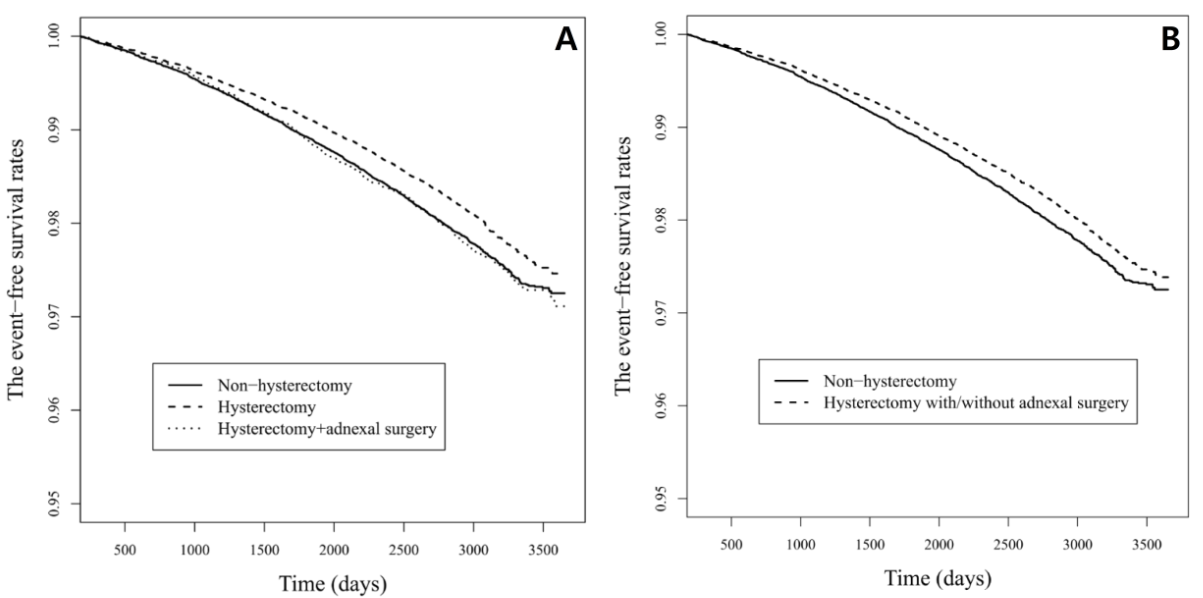

Supplement: Supplementary file 1 [file medicina-59-01627-s001.zip › Supplementary_Figure S1.png]
